# Supplementary material for: Causal Relationship between Aging and Anorexia Nervosa: A White-Matter-Microstructure-Mediated Mendelian Randomization Analysis
Source: Biomedicines. 2024 Aug 16;12(8):1874. doi: 10.3390/biomedicines12081874 (PMC11351342; doi:10.3390/biomedicines12081874)
Supplement: Supplementary file 1 [file biomedicines-12-01874-s001.zip › Supplementary Figure/Figure S4/Singe SNP plot of MR analysis for TL to ubm-b-1510.pdf]

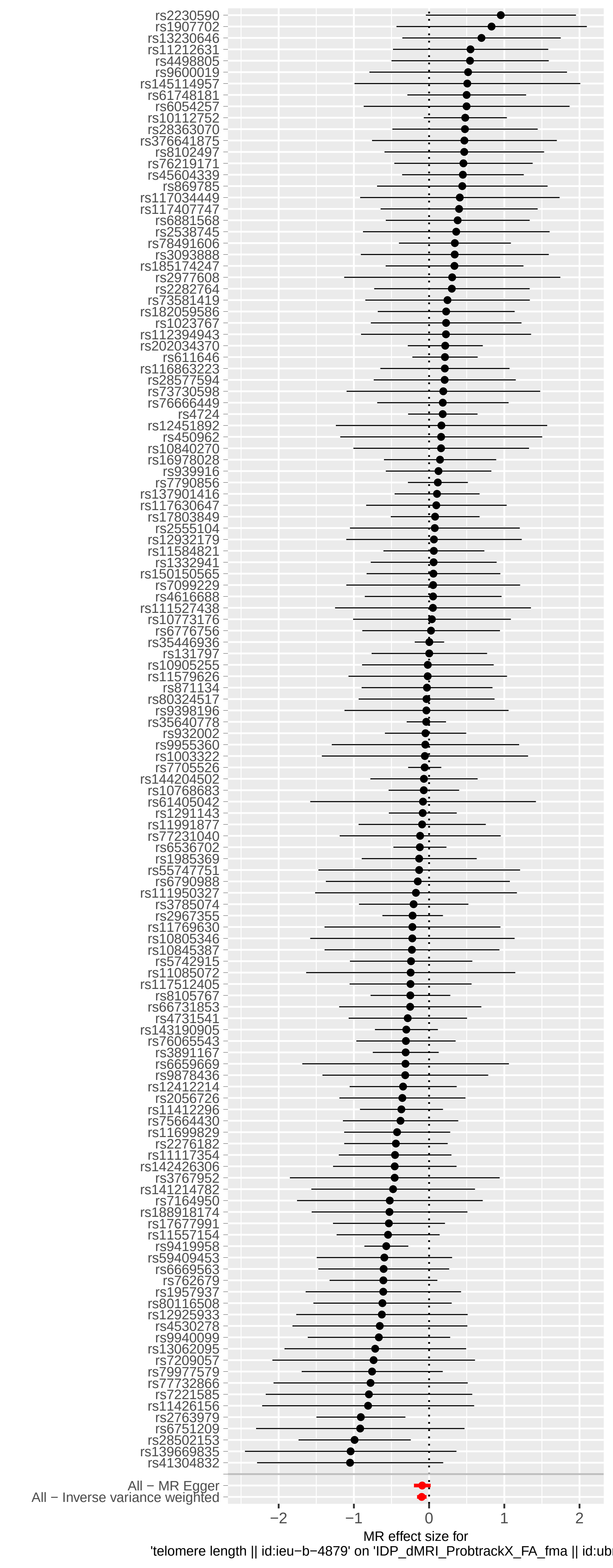
rs2230590
rs1907702
rs13230646
rs11212631
rs4498805
rs9600019
rs145114957
rs61748181
rs6054257
rs10112752
rs28363070
rs376641875
rs8102497
rs76219171
rs45604339
rs869785
rs117034449
rs117407747
rs6881568
rs2538745
rs78491606
rs3093888
rs185174247
rs2977608
rs2282764
rs73581419
rs182059586
rs1023767
rs112394943
rs202034370
rs611646
rs116863223
rs28577594
rs73730598
rs76666449
rs4724
rs12451892
rs450962
rs10840270
rs16978028
rs939916
rs7790856
rs137901416
rs117630647
rs17803849
rs2555104
rs12932179
rs11584821
rs1332941
rs150150565
rs7099229
rs4616688
rs111527438
rs10773176
rs6776756
rs35446936
rs131797
rs10905255
rs11579626
rs871134
rs80324517
rs9398196
rs35640778
rs932002
rs9955360
rs1003322
rs7705526
rs144204502
rs10768683
rs61405042
rs1291143
rs11991877
rs77231040
rs6536702
rs1985369
rs55747751
rs6790988
rs111950327
rs3785074
rs2967355
rs11769630
rs10805346
rs10845387
rs5742915
rs11085072
rs117512405
rs8105767
rs66731853
rs4731541
rs143190905
rs76065543
rs3891167
rs6659669
rs9878436
rs12412214
rs2056726
rs11412296
rs75664430
rs11699829
rs2276182
rs11117354
rs142426306
rs3767952
rs141214782
rs7164950
rs188918174
rs17677991
rs11557154
rs9419958
rs59409453
rs6669563
rs762679
rs1957937
rs80116508
rs12925933
rs4530278
rs9940099
rs13062095
rs7209057
rs79977579
rs77732866
rs7221585
rs11426156
rs2763979
rs6751209
rs28502153
rs139669835
rs41304832
All − MR Egger
All − Inverse variance weighted
−2
−1
0
1
2
MR effect size for
'telomere length || id:ieu−b−4879' on 'IDP_dMRI_ProbtrackX_FA_fma || id:ub
